# Momentary state anhedonia is associated with the quantity and quality of daily-life peer experiences among adolescents at varying risk for suicidal thoughts and behaviors

**DOI:** 10.1017/S0954579425100539

**Published:** 2025-08-26

**Authors:** Julianne M. Griffith, Margaret V. Brehm, Kiera M. James, Lori N. Scott, Caroline W. Oppenheimer, Cecile D. Ladouceur, Jennifer S. Silk

**Affiliations:** 1 Department of Psychiatry, University of Pittsburgh School of Medicine, Pittsburgh, PA, USA; 2 Department of Psychology, Univeristy of Pittsburgh, Pittsburgh, PA, USA; 3 Research Triangle Institute International, Research Triangle Park, NC, USA

**Keywords:** Adolescence, anhedonia, ecological momentary assessment, peer relationships

## Abstract

Anhedonia is a common and impairing symptom of psychopathology that predicts negative outcomes and may undermine peer relationships. Anhedonia comprises both *trait* (stable, time-invariant) and *state* (dynamic, time-varying) components. Relative to trait anhedonia, state anhedonia may be more strongly related to proximal risk for deleterious outcomes. Yet, associations between state anhedonia and daily-life socio-affective experiences in adolescence are not well understood. Thus, the present study used ecological momentary assessment (EMA) to examine within-person associations between state anhedonia and the quantity and quality of daily-life peer interactions among a sample of adolescents enriched for suicidality risk, a population at high risk for anhedonic and peer problems. Participants included 102 adolescents assigned female at birth (ages 12–18; M[SD] = 15.34[1.50]; 67.6% at elevated risk for suicidality). State anhedonia, as well as being with peers, connectedness with peers, and positive affect with peers, was measured three times per day for 10 days via EMA (*n* = 30 prompts). Multilevel models demonstrated that within-person fluctuations in state anhedonia relate to reduced odds of being with peers, as well as decreased connectedness and positive affect with peers. Findings suggest that dynamic changes in state anhedonia are related to both the *quantity* and *quality* of peer experiences among adolescents.

## Introduction

Anhedonia, or the loss of interest and/or pleasure in daily activities and experiences, is an important symptom of several forms of psychopathology including major depressive disorder, posttraumatic stress disorder, and transdiagnostic internalizing, and is associated with high levels of distress and functional impairment (Conway et al., 2019; Gabbay et al., 2015; Vinograd et al., 2022). Anhedonia may be especially influential during adolescence, a period during which risk for psychopathology rises, and long-term patterns of health and well-being are established (Paus et al., 2008; Sawyer et al., 2012). Specifically, anhedonia may interfere with adolescents’ ability to engage in normative approach-oriented behaviors critical to achieving key developmental milestones vital to a successful transition to adulthood, such as active participation in school and work and developing social consciousness and personal identity (Roisman et al., 2004; Schulenberg et al., 2004). Importantly, it may also interfere with the ability to cultivate meaningful peer relationships during a time when social salience is heightened and peer interactions are developmentally important (Somerville et al., 2013; Watson et al., 2020). For adolescents at increased risk for suicidal thoughts and behaviors (STBs), links between anhedonia and impairments in peer functioning may be particularly consequential. These impairments include both the frequency of interactions with peers and the social-affective quality of those interactions (e.g., connectedness, positive affect), which we refer to as the quantity and quality of peer experiences, respectively. That is, anhedonia may contribute to impairments in teens’ motivation to engage in peer interactions, as well as interfere with the subjective quality of those interactions, potentially exacerbating and reinforcing feelings of social disconnectedness, thwarted belongingness, and perceived burdensomeness, which are theorized to play a central role in the etiology of suicidal behavior (Auerbach et al., 2022; Van Orden et al., 2010).

Research on anhedonia in adolescents has predominantly relied on questionnaire measures that assess *trait* or relatively stable patterns of anhedonic features, rather than *state*, or momentary, dynamic levels of anhedonia as they are experienced by adolescents in daily life. It is known, however, that anhedonia is likely to fluctuate over the course of a day or week (see Auerbach et al., 2022 for further discussion). This is a critical gap, as trait and state anhedonia may relate to outcomes of interest in different ways (see e.g., Yang et al., 2020), with state anhedonia hypothesized to be a more salient predictor of proximal risk for deleterious outcomes, including STBs, than trait anhedonia. Understanding the behavioral and affective indicators and correlates of state anhedonia in daily-life peer contexts among vulnerable teens is vital to identifying mechanisms of risk, as well as informing just-in-time interventions to promote adolescent well-being prior to the onset of a suicidal crisis.

Thus, the present study used ecological momentary assessment (EMA), an ecologically valid tool for assessing momentary, real-life feelings and experiences (Shiffman et al., 2008), to investigate daily-life associations between state anhedonia and the quality and quantity of peer experiences among adolescents oversampled for STB risk. Specifically, this study examined associations between adolescent state anhedonic symptoms and (1) their likelihood of being in the company of peers, (2) their feelings of connectedness with peers, and (3) their subjective feelings of positive affect when in the company of peers.

### The importance of peer interactions during adolescence

During adolescence, peer relationships become increasingly salient in youths’ lives (Guyer et al., 2016). Amid cognitive, biological, and environmental changes (Somerville et al., 2013), teens begin to attend more toward their peer environment as they establish budding autonomy from parents (Bokhorst et al., 2010; Veenstra & Laninga-Wijnen, 2022). Establishing high-quality friendships during this time is developmentally important for future positive socioemotional, relational, and psychological outcomes (La Greca & Harrison, 2005; Parker et al., 2006). Additionally, peer interactions provide valuable affective benefits in the short term. For example, EMA studies have shown that social interactions are associated in real time with reduced state loneliness and improved mood (Silk et al., 2011; van Roekel et al., 2015; Walsh et al., 2024). Specifically, this work shows that adolescents report lower momentary sadness (Silk et al., 2011; Walsh et al., 2024) and state loneliness (van Roekel et al., 2015) when in the immediate company of others, broadly, and peers, specifically (Silk et al., 2011; van Roekel et al., 2015). These findings suggest that alongside larger social developmental processes, connectedness to peers, thought of as teens’ perception of closeness or belonging with peers, is associated with positive proximal outcomes for adolescents.

Indeed, positive peer interactions are particularly crucial for teens at high risk for STBs (Fredrick et al., 2018). Several theoretical frameworks converge to suggest that social-affective experiences in peer contexts, including frequency of peer interactions, positive affect with peers, and social connectedness, are influential to STB risk among vulnerable teens. According to the interpersonal theory of suicide, for example, individuals experiencing thwarted belongingness and perceived burdensomeness are at elevated risk for suicide (Van Orden et al., 2010). Feelings of thwarted belongingness, or the “unmet need to belong,” is a cognitive-affective state thought to arise from a lack of supportive relationships (i.e., low quantity of relationships) as well as the absence of fulfilling social interactions (i.e., low quality relationships). The Three-Step Theory of Suicide (3ST; Klonsky & May, 2015) also emphasizes the importance of positive peer experiences as a buffer against suicidal behavior, positing that high-quality social experiences serve as a powerful protective factor for those most at risk for escalations in suicidal ideation.

Empirical work supports associations between social connectedness and risk for escalations in STBs among adolescents (see e.g., Hutchinson et al., 2021; Mackin et al., 2017; Whitlock et al., 2014). For example, positive peer relationship quality was found to be protective against increases in suicidal ideation over time in a sample of 13–15 year-old adolescent girls (Mackin et al., 2017). Moreover, daily peer connectedness was found to associate with reduced odds of experiencing suicidal ideation during the COVID-19 pandemic in another study of 12–17 year-old girls (Hutchinson et al., 2021). Conversely, a wealth of research implicates peer adversity, including social rejection and conflict, in risk for STBs among young people (Nock et al., 2009; Oppenheimer et al., 2020; Victor et al., 2019; see Cheek et al., 2020 for review). Taken together, this body of research highlights the critical role that peer interactions play in both cultivating risk and promoting resilience among teens at enhanced risk for STBs.

### Anhedonia and adolescent peer functioning

Anhedonia is associated with a host of impairments relevant to social functioning, including reduced motivation, impaired reward learning, and behavioral withdrawal (Pizzagalli, 2014). In the context of peer interactions, this would suggest that adolescents experiencing anhedonia may be less motivated to seek out interactions with peers, less rewarded by positive peer experiences, and more inclined to opt out of potentially pleasurable peer events. Consistent with this hypothesis, research conducted among adults finds that anhedonia potentiates a “spiral of withdrawal” wherein avoidance contributes to increases in anhedonia, which then contributes to further increases in withdrawal (see Barkus, 2021). Moreover, qualitative research conducted among adolescents finds that youth describe their experience of anhedonia as comprising feelings of disconnectedness and loss of belongingness with others (Watson et al., 2020). This aligns with findings from neuroimaging research demonstrating associations between anhedonia and altered activity in neural reward circuitry in response to social reward in emerging adults (Healey et al., 2014). Of note, however, this work has predominantly relied on measures of trait anhedonia and global assessments of social functioning, leaving much to be learned regarding momentary associations between fluctuations in state anhedonia and specific social experiences relevant to proximal risk and resilience, such as peer interactions, among vulnerable adolescents.

Clarifying associations between state anhedonia and adolescent peer functioning is important, as some limited research suggests that social impairments associated with state anhedonia may be powerful predictors of deleterious outcomes, including suicidal ideation, in young people. In a sample of undergraduate students, for example, loss of interest in people over the past two weeks was found to directly associate with suicidal ideation, whereas trait social anhedonia (i.e., dispositional tendencies to enjoy social interactions) was not (Yang et al., 2020). Moreover, in a sample of adolescents, loss of interest in friends over the past week was found to associate with increased risk for suicidal ideation, above and beyond the effects of trait anhedonia (Yang et al., 2021). These findings suggest that fluctuations in anhedonia may predict proximal alterations in teen’s motivation for and experience of social interactions, with implications for health and well-being. Yet, patterns of associations between state anhedonia and the frequency and subjective socio-affective quality of adolescent peer experiences remain unknown.

### Limitations of previous research: the need for ambulatory assessment

Existing work provides strong evidence that anhedonia is associated with social impairments, broadly defined, and that such impairments may be particularly pronounced among individuals at risk for STBs (e.g., Auerbach et al., 2022; Barkus, 2021; Pizzagalli, 2014; Van Orden et al., 2010). Limited research, however, has specifically evaluated the function of anhedonia as it relates to peer experiences in *adolescence*, a vulnerable period for the onset of psychopathology (Paus et al., 2008), While providing an exciting start, the limited work that has examined state anhedonia in adolescence has relied upon questionnaire measures evaluating state anhedonia over the past week (Yang et al., 2021), which restricts insights into more momentary associations between anhedonia and real-life social functioning. Moreover, this work has not examined *to what extent* and *in what ways* state anhedonia may influence peer experiences, specifically, in adolescence, despite the uniquely important role of positive peer relationships in promoting resilience and scaffolding healthy adolescent development (Bokhorst et al., 2010; Griffith et al., 2021; Kochel et al., 2017; La Greca & Harrison, 2005; Moses & Villodas, 2017; Parker et al., 2006).

The use of ambulatory assessment technology, including EMA, provides unique insights into adolescents’ daily-life moods and peer experiences as they unfold in naturalistic settings (Russell & Gajos, 2020; Shiffman et al., 2008). EMA is ideally suited to capture momentary fluctuations in state anhedonia over the course of a day, yielding fine-grained insights into teens’ dynamic experience of daily-life anhedonic mood that cannot be adequately measured using traditional questionnaire measures. Moreover, EMA has been fruitfully applied to advance understanding of adolescents’ everyday peer experiences, providing precise, ecologically valid assessments of the frequency and perceived quality of adolescents’ interactions with same-aged peers that are unaffected by limitations characteristic of traditional questionnaire measures, such as retrospective recall bias (see e.g., Alarcón et al., 2020; James, Sequeira et al., 2023; Sequeira et al., 2021; Sequeira, Griffith et al., 2024; Shiffman et al., 2008). Thus, EMA holds unique promise for illuminating momentary associations between adolescent fluctuations in state anhedonia and their daily-life peer experiences, which may help to clarify risk pathways linking anhedonia with proximal deteriorations in well-being and ultimately inform just-in-time interventions to circumvent risk and bolster vulnerable adolescents’ socioemotional health.

### Focusing on adolescent girls

Previous research indicates that compared to adolescent boys, adolescent girls are at increased risk for experiencing the onset of affective disorders and STBs during the adolescent transition (Hankin et al., 1998, 2015; Huang et al., 2017; Miranda-Mendizabal et al., 2019). Adolescent girls also demonstrate greater orientation toward and sensitivity to interpersonal feedback relative to adolescent boys (Rose & Rudolph, 2006; Rudolph, 2002). This suggests that adolescent girls may demonstrate more variability in state anhedonia relative to adolescent boys, and that associations between state anhedonia and peer experiences may be especially consequential in this population. That is, given girls’ greater normative investment in peer relationships, ties between mood and deteriorations in peer functioning have the potential to be self-reinforcing, and such associations may comprise an especially salient contributor to cascades of psychosocial risk among vulnerable adolescents. Thus, research is needed investigating associations between state anhedonia and peer experiences in adolescent girls, specifically.

### The current study

To address gaps in the existing literature, the present work leveraged smartphone-based EMA to examine proximal associations between within-person fluctuations in state anhedonia and (1) the likelihood of adolescents engaging in-person interactions with peers, (2) adolescents’ subjective feelings of connectedness with peers, and (3) adolescents’ subjective feelings of positive affect when in the company of peers. Given evidence that adolescents at risk for STBs may be particularly vulnerable to aversive peer experiences (Nock et al., 2009; Oppenheimer et al., 2020; Victor et al., 2019; see Cheek et al., 2020), the present study also examined whether associations between state anhedonia and daily-life peer experiences were moderated by adolescents’ baseline levels of suicidal ideation. We focused on in-person peer experiences, specifically, as a rich literature demonstrates the importance of in-person peer experiences for adolescent social-affective development, and it is as of yet unclear exactly how digitally-mediated interactions with peers (e.g., via text, social media, etc.) may vary from in-person experiences in terms of function and implications for risk and resilience. Taken together, study aims endeavored to characterize associations between fluctuating experiences of anhedonia and daily-life peer experiences as they unfold in the real-world, providing nuanced insights into how and in what ways state anhedonia relates to peer impairments in vulnerable teens.

We hypothesized that state anhedonia would be negatively related to (1) the likelihood that adolescents would report being in the presence of peers, (2) feelings of connectedness with peers, and (3) positive affect when in the company of peers. We further hypothesized that baseline suicidal ideation would moderate associations such that negative associations between anhedonia and these different facets of peer experiences would be stronger among adolescents with higher levels of baseline suicidal ideation.

## Methods

### Participants and procedures

Participants included 102 adolescents assigned female at birth (ages 12–18; *M* = 15.34, SD = 1.50). Adolescents were oversampled for suicidality risk such that 67.6% (n = 69) adolescents were at high risk for STBs as determined based on (a) recurrent suicidal ideation within the past 6 weeks, (b) ≥ 5 instances of non-suicidal self-injury within the past year, and/or (c) ≥1 suicide attempt within the past year. That is, teens were categorized as “high risk” if they endorsed one or more of these risk criteria. Teens were categorized as “low risk” if they denied a lifetime history of self-injurious thoughts and behaviors. In terms of gender, 76.5% of participants identified as girls, and 19.6% identified as transgender, nonbinary, or other gender minority.^1^ In terms of race, 2.0% of the sample identified as Asian/Asian American, 3.1% Biracial, 6.9% Black, 86.3% white, and 1% of another racial background. 3.0% of the sample identified as Hispanic/Latine. Median parent-reported family income before taxes was $90,001–100,000. Participants were recruited from the general community and outpatient clinics through flyers, internet advertisements, and the University of Pittsburgh research portal. Inclusion criteria included female sex assigned at birth and access to a personal smartphone. Exclusion criteria included lifetime presence of a neurological or serious medical condition, or psychotic, autism spectrum, or severe current substance use disorder. Presence of MRI contraindications were also exclusionary given broader study aims.

All study procedures were approved by the Institutional Review Board prior to data collection. Interested participants completed an initial phone screen in which eligibility criteria and history of STBs were assessed. Eligible participants were then invited to complete an initial baseline visit during which parents and adolescents were provided detailed information regarding study protocols and procedures, written informed parental consent and adolescent assent were obtained, and eligibility criteria and risk status (high versus low) were confirmed using the Kiddie Schedule for Affective Disorders and Schizophrenia – Present and Lifetime Version (KSADS-PL; Kaufman et al., 1997) and Self-Injurious Thoughts and Behavior Interview (SITBI; Nock et al., 2007). Participants also completed a brief demographics survey assessing gender and racial identities, as well as the Suicidal Ideation Questionnaire - Jr (SIQ-Jr; Reynolds, 1987). Approximately five weeks following this initial baseline assessment, teens completed a 10-day smartphone-based EMA procedure assessing momentary feelings and peer experiences.^2^ Specifically, teens were assisted with accessing a secure Web Data Express (WDX) web portal designed for the completion of EMA surveys on their personal smartphone devices, and research staff led participants through a sample survey, providing detailed information regarding the study protocol and clarifying any potential points of confusion. Teens were encouraged to ask questions throughout the training to ensure understanding. Staff emphasized the importance of completing surveys independently and instructed teens to keep all survey responses private. Adolescents were then prompted to respond to brief EMA surveys at three semi-random times outside of school hours (morning/before school, afternoon/after school, evening) each day for 10 days using the WDX portal (n = 30 total EMAs). Adolescents were permitted to select the time at which their morning survey arrived within a 30-minute window. Afternoon surveys were delivered at random times between 3:30–6:30 pm, and evening surveys were delivered at random times between 6:30–9:30 pm. Adolescents had 60 minutes to complete each survey before it expired. Survey links were delivered via text message, and teens received reminder texts every 15 minutes until the survey was completed or expired (*n* = 3 possible reminders). EMA completion was routinely monitored by study staff at least three times per week, and participants with multiple missing surveys were prompted via text message to troubleshoot any technical problems and encourage EMA completion. Participants completed an average of 77% of surveys (SD = 18%, range = 17%−100%).^3^ EMA completion rate was positively correlated with age (*r* = .20, *p* = .042), such that older adolescents had higher completion rates relative to younger adolescents. EMA completion rate was not correlated with baseline suicidal ideation (*r* = −.17, *p* = .104). Adolescents received monetary compensation in recognition of their participation. Specifically, adolescents were compensated according to the number of EMA surveys completed such that teens earned $30 for completing 9–15 surveys, $50 for completing 16–20 surveys, and $80 for completing 21–30 surveys. Adolescents who completed fewer than 9 surveys were not provided with compensation but were offered the opportunity to restart the full EMA protocol the following week.

### Measures

#### State anhedonia

State anhedonia was assessed on each EMA survey using an item from an EMA-adapted version of the Patient Health Questionnaire – 9 (PHQ-9; Kroenke et al., 2001). Specifically, participants were asked to indicate how much they were bothered since the last EMA survey (with a reminder of the last survey time) by “little interest or pleasure in doing things” on a 4-point Likert-style scale from 1 (*not at all*) to 4 (*most of the time*). The PHQ-9 is a valid and reliable measure that has been used in prior EMA studies (Torous et al., 2015).

### Being with peers

Being in the company of peers (0=not with peers, 1=with peers) was assessed at each EMA survey. Specifically, at each survey, adolescents were asked to indicate who they were with in person when the survey was received from a predetermined series of options. Teens were coded as being with peers if they indicated currently being in the presence of friends, romantic partners, or other teens (e.g., classmates, teammates). Online interactions were considered beyond the focus of the present study.

### Connectedness with peers

Connectedness with peers was also assessed using EMA. Participants who reported currently being in the presence of peers were instructed to rate how close and connected they felt with those peers at the time of the alert on a scale from 0 *(not at all)* to 100 *(extremely).* Participants who reported currently being with multiple types of peers (close friends, other friends, romantic partners, or other teens) were prompted to provide a connectedness rating for each, and scores were averaged to obtain a rating of current connectedness with peers.

### PA with peers

PA with peers was similarly assessed using EMA. At the beginning of each EMA survey (prior to assessing social context), participants were prompted to rate the extent to which they were currently feeling different emotion states using a 0 (*not at all*) to 100 (*extremely)* sliding Visual Analog Scale (VAS). For the purposes of the present analyses, PA with peers was calculated as the average of adolescent momentary “happy,” “joyful,” “interested,” and “excited” emotions on EMA surveys in which teens subsequently endorsed being with peers, as defined above.

### Suicidal ideation

Suicidal ideation was assessed at baseline using the SIQ-Jr (Reynolds, 1987). The SIQ-Jr comprises 15 questions assessing a range of suicidal ideations (e.g., “I thought it would be better if I was not alive,” “I wished I were dead,” “I thought that killing myself would solve my problems”). For each item, participants were prompted to rate how often they experienced each thought over the past month from 0 (*I never had this thought*) to 6 (*almost every day*). Items are summed to yield a total score with a range of 0–90, with higher scores indicating greater levels of suicidal ideation. Internal consistency in the present study was excellent (ω =.97).

### Data analytic plan

Hypotheses were tested using a series of multilevel models implemented using the “lme4” package in R (Bates et al., 2015; R Core Team, 2022). To evaluate associations between state anhedonia and odds of being with peers, we conducted a multilevel logistic regression in which peer engagement status (0=not with peers, 1=with peers) was regressed on state anhedonia. Next, we evaluated associations between state anhedonia and feelings of connectedness and PA while with peers using a series of multilevel models using restricted likelihood estimation (REML). For these analyses, we included only those surveys in which adolescents endorsed being in the presence of peers (n = 585; 24.4% of completed surveys). Finally, we examined cross-level interactions between baseline suicidal ideation and state anhedonia on peer outcomes. Age was included as a covariate in all analyses. Person-mean anhedonia scores (i.e., adolescents’ average anhedonia ratings across EMAs) representing individual differences in mean anhedonia (“trait anhedonia”) were also entered as a covariate. Level 1 continuous predictors (i.e., state anhedonia) were person mean-centered and Level 2 predictors and covariates (i.e., suicidal ideation, age, person-mean anhedonia) were grand mean-centered prior to analysis. For all analyses, effect sizes were calculated using “EMAtools” (Kleiman, 2017).

## Results

### Preliminary analyses

Descriptive statistics and bivariate correlations characterizing primary variables of interest are reported in Table 1. Anhedonia was negatively correlated with average connectedness (*r* = −.37) and PA when with peers (*r* = −.28). Anhedonia was positively correlated with baseline suicidal ideation (*r* = .56).

### Tests of primary hypotheses

Complete results of multilevel models are reported in Table 2. Findings indicated that state anhedonia was significantly negatively related to adolescent odds of being with peers (*b* = −.14, *p* < .001, OR = .66). Multilevel models also revealed significant negative associations between state anhedonia and momentary feelings of connectedness with peers (*b* = −11.16, *p* < .001, Cohen’s *d* = −1.86), such that on occasions in which adolescents felt more anhedonic, they reported feeling less connected with peers. State anhedonia was also negatively associated with momentary PA with peers (*b* = −10.08, *p* < .001, Cohen’s *d* = −1.70); on occasions in which adolescents felt more anhedonic, they reported lower levels of PA when in the presence of peers.

**Figure 1.**
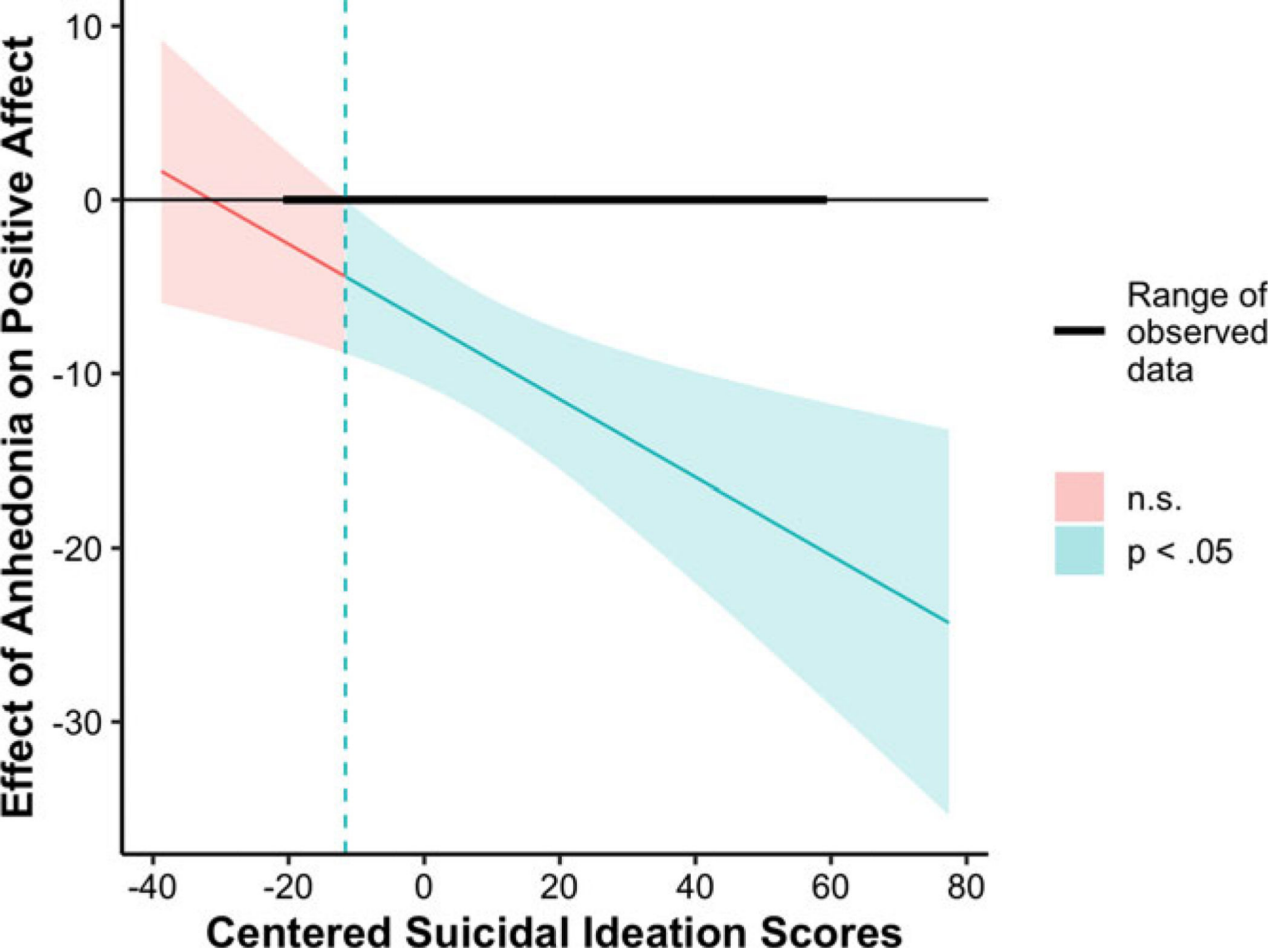
Johnson-Neyman plot visualizing the strength of association between state anhedonia and positive affect with peers at different levels of baseline suicidal ideation.

Results of cross-level interaction models are reported in Table 3. Main effects of state anhedonia were retained across outcomes. Suicidal ideation did not moderate associations between state anhedonia and odds of being with peers or feelings of connectedness with peers. Suicidal ideation interacted with state anhedonia to predict positive affect with peers (*b* = −.22, SE = .08, *p* = .003). Further examination using the Johnson-Neyman technique implemented using the “jtools” package (Long, 2022) indicated that the magnitude of the negative association between state anhedonia and positive affect with peers was larger among adolescents at higher levels of suicidal ideation. At low levels of baseline suicidal ideation (<8.57 on the SIQ), state anhedonia was no longer a significant predictor of positive affect with peers (see Figure 1).

### Exploratory analyses

To further evaluate the timescale and temporal sequencing of associations between state anhedonia and adolescent peer experiences, exploratory models were also conducted examining prospective associations between average state anhedonia on day *t* and adolescent (1) odds of being with peers at any point on day *t* + *1*, (2) mean connectedness with peers on day *t* + *1*, and (3) mean positive affect when with peers at day *t* + *1*.^4^ No significant associations between average state anhedonia and next-day peer experiences were found (see Table 4).

## Discussion

Using a risk-enhanced sample comprising teens at elevated risk for STBs and an EMA design, the present study examined real-life associations between within-person fluctuations in state anhedonia and the quantity (i.e., likelihood of being with peers) and quality (i.e., feelings of connectedness and PA) of peer experiences in adolescent girls. Findings reveal that when adolescents experience momentary increases in anhedonia, they are less likely to be in the company of peers *and* they derive less connectedness from their peer experiences. Moreover, suicidal ideation was found to moderate associations between state anhedonia and positive affect with peers such that negative effects of state anhedonia on positive affect with peers are larger among teens experiencing greater suicidal ideation. Associations between state anhedonia and impairments and the quantity and quality of peer experiences were observed regardless of teens’ levels of trait anhedonia. Together, results indicate that among teens with varying levels of suicidal ideation, momentary anhedonia is related to lower *quantity* and *quality* of their peer experiences. Findings indicate that state anhedonia may be implicated in cycles of withdrawal and social disconnectedness in vulnerable teens, with implications for understanding daily-life mechanisms of risk for cascading socio-affective dysfunction among girls at increased risk for STBs.

Results of the present work demonstrate that momentary increases in state anhedonia are associated with reduced odds that adolescents will spend time in the company of peers. This finding is consistent with research in adults indicating that anhedonia potentiates a “spiral of withdrawal” from social interactions (Barkus, 2021). For example, studies conducted with college-aged individuals have shown that, when experiencing high levels of anhedonia, emerging adults spend more time alone by choice (Brown et al., 2007; Kwapil et al., 2009). Results are also consistent with theoretical models of anhedonia, which posit that anhedonic symptoms impair motivation and interfere with reinforcement learning processes that might otherwise promote peer engagement during adolescence (Pizzagalli, 2014). Future research may wish to directly assess social motivation, in addition to frequency of being with peers, to identify mechanisms linking elevations in state anhedonia to peer withdrawal among high-risk teens.

Findings also indicate thatwithin-person increases in anhedonia are related to lower feelings of connectedness with peers across our sample of adolescents, as well as lower positive affect when in the company of peers among adolescents experiencing suicidal ideation at baseline. Findings align with qualitative work indicating that decreases in peer connectedness comprise a highly salient and impairing characteristic of adolescent anhedonia (Watson et al., 2020), and extend this work to show that dynamic within-person fluctuations in state anhedonia relate to corresponding contemporaneous impairments in high-risk teens’ ability to connect and derive pleasure from peer experiences in everyday settings. Indeed, when in the presence of peers, individuals experiencing suicidal ideation may be particularly sensitive to variation in momentary anhedonia, resulting in less engaged, social, and affiliative feelings with peers (Brown et al., 2007; Kwapil et al., 2009; Llerena et al., 2012). In this way, anhedonia may decrease the subjective rewarding nature of peer interactions, but it also may lead to less affiliative behaviors with others, such as withdrawing or disengaging, which could further decrease the quality of peer interactions.

Given the key role of positive peer relationships in bolstering adolescent resilience and promoting positive psychosocial outcomes across both micro- (Sequeira, Griffith et al., 2024) and macro-level timescales (Bokhorst et al., 2010; Griffith et al., 2021; Kochel et al., 2017; La Greca & Harrison, 2005; Moses & Villodas, 2017; Parker et al., 2006), this pattern of findings suggest that one important way in which anhedonia exerts its pernicious effects may be through its associations with adolescents’ capacity to enjoy the benefits of everyday moments of pleasure with friends, romantic partners, and other same-aged teens. Over time, deficits in such momentary, daily-life experiences of connectedness and positive affect may reinforce depressive psychopathology and STBs, and crystallize into more enduring feelings of social disconnection. In this way, state anhedonia-peer functioning associations may contribute to a cascading pattern of risk, with downstream implications for suicidal thoughts and behaviors (Van Orden et al., 2010). More broadly, this finding is consequential, as adolescence is a key period of socio-affective learning (Crone & Dahl, 2012; Guyer et al., 2016), and impairments in teens’ ability to connect and experience mutual joy with peers may impact the formation of long-term friendships that might otherwise scaffold the transition to emerging adulthood.

Contrary to hypotheses, associations between state anhedonia and odds of being with peers or feelings of connectedness with peers were not moderated by baseline suicidal ideation. That is, greater state anhedonia was associated with likelihood of adolescents interacting with peers, as well as lower connectedness with peers regardless of teens’ level of suicidality. Given the characteristics of the present sample, which was enriched for adolescents at elevated risk for STBs, this finding suggests that momentary fluctuations in anhedonic mood are consequential and meaningful correlates of psychosocial functioning among adolescents with and without suicidality. Careful monitoring of daily changes in state anhedonia may, therefore, be useful for detecting risk for negative peer outcomes for adolescents experiencing a range of STBs.

Of note, exploratory analyses evaluating day-to-day prospective associations between average state anhedonia and next-day peer experiences indicated no significant relations between anhedonia on day *t* and teens’ likelihood of being with peers or feelings of connectedness or positive affect with peers on day *t* + *1*. This may suggest that associations between state anhedonia and adolescents’ peer experiences unfold across finer-grained timescales (e.g., minute-to-minute, hour-to-hour) than were captured in the present study design. This is consistent with conceptualizations of state anhedonia as dynamic in nature, and it is likely that momentary fluctuations in state anhedonia are not well-represented using day-level aggregates. Further research is needed using more temporally sensitive EMA designs to better capture rapid changes in mood and social functioning to clarify the timescale and sequencing of effects.

The present work benefits from several strengths that advance the existing literature and bolster confidence in findings. First, the present use of EMA provides new insights into daily-life changes in state anhedonia, and yields novel information about within-persons associations between state anhedonia and peer functioning as they unfold in adolescents’ naturalistic contexts. This significantly extends previous research, which has predominantly relied upon questionnaire measures and/or assessments of trait anhedonia (Auerbach et al., 2022; Yang et al., 2020, 2021). Additionally, the present work included a sample of adolescents at elevated risk for STBs, as defined by recent instances of suicidal ideation, non-suicidal self-injury, and/or suicide attempts. Given the role of interpersonal factors in risk for suicide (Klonsky & May, 2015; Van Orden et al., 2010), the characteristics of the present sample allowed for rigorous analysis of anhedonia-peer functioning associations in highly vulnerable teens. Finally, the present study evaluated associations between state anhedonia and both the *quantity* and *quality* of peer experiences, illuminating specific, nuanced ways in which fluctuations in momentary anhedonic mood influence different aspects of adolescent peer functioning in daily life.

Results should also be interpreted considering several limitations that represent important areas for future research. First, the present study relied on a single-item measure of state anhedonia. Single-item measures EMA have been found to be valid and reliable in previous research (Song et al., 2023); however, future work may wish to incorporate multi-item measures that capture differing dimensions of anhedonia (e.g., social, physical). Moreover, state anhedonia was assessed “since the last survey,” which may reduce the temporal precision of the measure. Future work should measure anhedonia *in the moment* to more precisely capture state anhedonic experience. Additionally, the present study design was correlational in nature and relied on adolescent self-report. To build on these findings, researchers may wish to incorporate other ambulatory assessment techniques to assess facets of daily-life peer functioning (e.g., passive digital monitoring), as well as more temporally sensitive study designs to clarify the time-course and sequencing of associations between state anhedonia and impairments in adolescent daily-life peer experiences. Additionally, the present work focused on in-person peer experiences, and it is likely that some adolescents engage in meaningful online peer interactions that were not measured in this study. Future work should build on the present findings and assess both in-person and online peer experiences to provide greater nuance into adolescent peer experiences across social contexts. Future work may also benefit from analyzing teens’ *motivation* to engage in peer interactions, in addition to adolescent odds of being with peers, as well as assessing other facets of teens’ social experiences including the activities they are engaging in with peers and the quality and quantity of non-peer social interactions. Further, additional research is needed to evaluate associations between other salient transdiagnostic symptoms of psychopathology common among adolescents (e.g., low mood, sleep disturbances, irritability) and daily-life peer outcomes.

Additional limitations related to the design of the present work are also worth noting. While the present EMA provides rich insights into adolescents’ experience *in context*, it is possible that ratings of connectedness and positive affect were affected by social desirability bias when teens were with peers, although the chances of social desirability effects were reduced through careful training encouraging teens to keep their responses private. Additionally, the extent to which teens’ mood was affected by non-peer social company (e.g., parents, siblings) is unclear. Additionally, due to logistical constraints, suicidal ideation was assessed five weeks prior to daily-life peer experiences, and it is possible that adolescents’ levels of suicidal ideation fluctuated in meaningful ways between baseline and initiation of the EMA. Moreover, the present sample was limited to adolescents assigned female at birth. Approximately 23% of participants identified as transgender, nonbinary, or another gender minority, representing a strength of the present study; however, further work is needed to evaluate the extent to which results generalize across different sex and gender groups (e.g., teens assigned male at birth). The present work was underpowered to evaluate gender effects, and future work should investigate how associations may vary across cisgender and gender-minority adolescents. Further, although the use of multilevel models leverages the strengths of dense repeated-measures EMA assessments to maximize power and yield robust estimates under conditions of limited sample size (Maas & Hox, 2005), no a priori power analyses were conducted, and it was possible that the study was not sufficiently powered to detect moderation effects by suicidal ideation, though note that one moderation effect was observed. Thus, it is important that results be replicated in larger samples, including a priori power analyses, to increase confidence in findings. Finally, participants predominantly identified as non-Hispanic/Latine and white. Future studies should aim to include adolescents across diverse racial and social identity groups to be appropriately representative and maximize generalizability of findings.

In summary, the present study used a novel EMA approach to evaluate momentary, within-person associations between state anhedonia and the quantity and quality of in-person peer experiences in adolescents at high and low risk for STBs. Findings reveal that fluctuations in anhedonic mood are related to lower quantity and quality of peer experiences, such that adolescents engage with peers less frequently and feel less connectedness with peers when experiencing elevations in state anhedonia, regardless of their baseline levels of suicidal ideation. For teens who have recently experienced elevated suicidal ideation, state anhedonia also impairs the pleasure derived from peer experiences in daily-life. Results demonstrate the salience of state anhedonia for understanding adolescents’ real-life peer functioning, with implications for understanding risk and resilience across development.

## Figures and Tables

**Table 1.** Descriptive statistics and bivariate correlations for primary variables of interest

	M(SD)	ICC	1.	2.	3.	4.
1. Anhedonia	1.55 (.68)	.61				
2. Connectedness with peers	70.11 (21.44)	.37	**−.37**			
3. PA with peers	64.39 (16.90)	.35	**−.28**	**.63**		
4. SIQ	20.19 (20.34)	–	**.56**	−.16	−.07	
5. Age	15.34 (1.50)	–	−.07	−.01	−.17	.06

*Note.* For the purposes of computing descriptive statistics and correlations, EMA variables have been averaged across sampling occasions per person. For this reason, we refer to the aggregate of individual state anhedonia assessments as “Anhedonia” for accuracy, as this measure no longer retains its state-level structure. Bolded values are significantly different from zero at *p* < .05. *M* = mean; SD = standard deviation; ICC = intraclass correlation coefficient; PA = positive affect; SIQ = Suicidal Ideation Questionnaire.

**Table 2.** Results of multilevel models evaluating associations between state anhedonia and daily-life experiences with peers

	*b*	SE(*b*)	*p*	OR
**Model 1: State anhedonia predicting odds of being with peers**				
Intercept	−1.52	.13	< .001	
State anhedonia	−.41	.11	< .001	.66
Trait anhedonia	−.17	.20	.401	.85
Age	.13	.09	.150	1.13
	*b*	SE(*b*)	*p*	Cohen’s *d*
**Model 2: State anhedonia predicting connectedness with peers**				
Intercept	68.30	1.86	< .001	
State anhedonia	−11.16	2.37	< .001	−1.86
Trait anhedonia	−13.56	2.83	< .001	−1.04
Age	−.07	1.21	.957	−.01
	*b*	SE(*b*)	*p*	Cohen’s *d*
**Model 3: State anhedonia predicting positive affect with peers**				
Intercept	62.72	1.54	< .001	
State anhedonia	−10.08	1.70	< .001	−.51
Trait anhedonia	−9.06	2.34	< .001	−.82
Age	−2.02	1.01	.049	−.46

*Note.* Trait anhedonia is represented using person-level mean anhedonia across EMAs. Cohen’s *d* values were calculated using ‘EMAtools’ (Kleiman, 2017). *b* = unstandardized effect; SE(*b*)=standard error of the unstandardized effect; OR = odds ratio.

**Table 3.** Results of cross-level interaction models evaluating moderation by suicidal ideation

	*b*	SE(*b*)	*p*	OR
**Model 1: State anhedonia × SIQ predicting odds of being with peers**					
Intercept	−1.56	.14	< .001	
State anhedonia	−.36	.13	.004	.70
SIQ	−.01	.01	.398	.99
State anhedonia × SIQ	−.01	.01	.307	.99
Trait anhedonia	−.08	.24	.733	.92
Age	.11	.09	.204	1.12
	*b*	SE(*b*)	*p*	Cohen’s *d*
**Model 2: State anhedonia × SIQ predicting connectedness with peers**					
Intercept	68.98	1.88	< .001	
State anhedonia	−9.97	2.23	< .001	−.41
SIQ	−.001	.11	.992	−.002
State anhedonia × SIQ	−.11	.09	.218	−.11
Trait anhedonia	−13.40	3.41	< .001	−.83
Age	−.48	1.23	.697	−.09
	*b*	SE(*b*)	*p*	Cohen’s *d*
**Model 3: State anhedonia × SIQ predicting positive affect with peers**					
Intercept	63.41	1.47	< .001	
State anhedonia	−7.02	1.85	< .001	−.34
SIQ	.03	.09	.621	.10
State anhedonia × SIQ	−.22	.08	.003	−.77
Trait anhedonia	−10.18	2.69	< .001	−.26
Age	−2.46	.97	.013	−.60

*Note.* Trait anhedonia is represented using person-level mean anhedonia across EMAs. Cohen’s *d* values were calculated using ‘EMAtools’ (Kleiman, 2017). *b* = unstandardized effect; SE(*b*)=standard error of the unstandardized effect; OR = odds ratio; SIQ = suicidal ideation questionnaire.

**Table 4.** Results of exploratory analyses examining associations between state anhedonia on day t and peer experiences on day t + 1

	*b*	SE(*b*)	*p*	OR
**Model 1: State anhedonia predicting next-day odds of being with peers**				
Intercept	−.47	.16	.003	
State anhedonia	.005	.21	.980	1.01
Trait anhedonia	−.12	.23	.601	.89
Age	.12	.10	.236	1.13
	*b*	SE(*b*)	*p*	Cohen’s *d*
**Model 2: State anhedonia predicting next-day connectedness with peers**				
Intercept	70.00	2.00	< .001	
State anhedonia	.42	3.29	.898	.01
Trait anhedonia	−13.67	3.03	< .001	−1.00
Age	.06	1.31	.961	.01
	*b*	SE(*b*)	*p*	Cohen’s *d*
**Model 3: State anhedonia predicting next-day positive affect with peers**				
Intercept	63.83	1.64	< .001	
State anhedonia	3.41	2.76	.217	.14
Trait anhedonia	−8.57	2.49	< .001	−.72
Age	−1.86	1.08	.088	−.39

*Note.* Trait anhedonia is represented using person-level mean anhedonia across EMAs. Cohen’s *d* values were calculated using ‘EMAtools’ (Kleiman, 2017). *b* = unstandardized effect; SE(*b*)=standard error of the unstandardized effect; OR = odds ratio.

## Data Availability

Data will be publicly available through the NIH Data Archive.

## Footnotes

1 A total of 3.9% of participants preferred not to disclose their gender.

2 In order to minimize participant burden given the potentially lengthy nature of clinical interview measures, the initial baseline visit was conducted virtually. Eligible participants were subsequently invited to complete an in-person follow-up visit during which they were assisted with EMA set-up and training. This second visit, which was required to be completed prior to initiation of the EMA, was conducted approximately five weeks after the initial baseline visit due to staffing and scheduling constraints.

3 Surveys were considered “completed” if participants successfully responded to all survey items.

4 Data were aggregated on the day level for ease of interpretation, given that long and unequal amounts of time elapsed between individual surveys within a given day. Moreover, state anhedonia and peer experiences were not assessed during the school day to avoid interfering with teens’ academic schedule, which may obscure key information regarding associations between state anhedonia and adolescent peer experiences in this highly salient developmental context and complicate interpretations of results.
